# Anti-mattering mediates the relationship between social-responsibility misalignments and mental health problems in young people

**DOI:** 10.3389/fpsyt.2025.1639802

**Published:** 2025-10-15

**Authors:** Patricia Gooding, Karolina Kluk-de Kort, Piers Ramsdale-Capper, Tracy Epton

**Affiliations:** ^1^ Division of Psychology and Mental Health, School of Health Sciences, Manchester Academic Health Sciences Centre, University of Manchester, Manchester, United Kingdom; ^2^ Division of Psychology, Communication and Human Neuroscience, School of Health Sciences, Manchester Academic Health Sciences Centre, University of Manchester, Manchester, United Kingdom

**Keywords:** mattering, social-responsibility, mental health, depression, anxiety, stress

## Abstract

**Background/objectives:**

Mental health problems among university students are increasing in prevalence, and it is vital to understand why. The detrimental effects of misalignments between corporate social-responsibility values and those of employees have been widely evidenced. We investigated how misalignments between the personal importance of social-responsibility values held by students versus those of their university affected their mental health. It was predicted that anti-mattering would mediate relationships between misalignments in social-responsibility values and mental health problems.

**Methods:**

Student participants (N=171) completed an online survey assessing the personal importance of nine social-responsibility domains together with the perceived importance of these domains to the student’s university. Participants also completed a measure of anti-mattering which assesses perceptions of being insignificant and invisible, and a composite measure of depression-anxiety-stress. Direct and indirect pathways were assessed with linear regression models.

**Results:**

There were four key findings. First, across the nine social-responsibility domains, personal importance ratings were significantly higher than those ascribed to the university. Second, misalignments in social-responsibility importance ratings were significantly associated with depression-anxiety-stress scores. Third, the relationship between the discrepancy in social-responsibility importance ratings and depression-anxiety-stress was mediated by anti-mattering. Fourth, the key characteristic of anti-mattering in this mediated pathway was perceived invisibility.

**Conclusion:**

There is potential for a positive effect on mental health to be gained if institutions, such as universities, authentically co-develop, instantiate, and evaluate social-responsibility values with stakeholders in ways that genuinely combat invisibility, and instead, reflect that the views and feelings of stakeholders do matter.

## 1 Introduction

A robust and expanding literature evidences the severity of mental health problems, especially depression and anxiety, faced by young people, including university and college students (1–3). The importance of studying mattering and not mattering to better understand mental health has been emphasised in both young people and adults (4–14) including via a recent review article (15). Conceptually, mattering arose from sociological and positive psychology approaches and has both experiential impact and motivational functions (16). Mattering comprises perceptions of being of value to others; feeling significant or important; feeling cared for; being needed by others; and feeling appreciated (6, 17, 18). In contrast, not mattering (19) or anti-mattering, reflects enduring and pervasive feelings and perceptions of being unimportant, marginalised, unvalued, invisible, insignificant, and inconsequential to others which become internalised (20, 21). Anti-mattering is considered to reflect more than the polar opposite of mattering. Rather it represents distinct dimensions which signal vulnerabilities to mental health problems (22). Indeed, the central role of anti-mattering in a number of mental health problems has been evidenced across numerous contexts and samples (23–27), including in depression and anxiety in students (4). Furthermore, anti-mattering has been shown to act as a mediator in pathways to depression and anxiety from perfectionism in students (28), and between anxiety and burnout during the Covid-19 pandemic, also in students (29). Examining anti-mattering as a mediator in pathways to depression, anxiety, and stress in university students was central to the current study.

The concept of social-responsibility incorporates a sense of personal and collective responsibility to ensure social justice and the actualisation of equal opportunities across diverse communities that make a difference to society (30, 31). Many organisations, both large and small, promote social-responsibility at the core of their business models (32). Organisations with a focus on social-responsibility include universities. However, the grounding tenets of social-responsibility in universities often differ from those commonly found in corporate social-responsibility agendas in that universities can transcend corporate business models to evaluate and instantiate social-responsibility frameworks that encourage innovation, engagement, and scrutiny in ways that span the boundaries of science, business, industry, and policy (33). Furthermore, it has been argued that the role of universities in shaping social-responsibility agendas extends beyond the traditional principles of discovery and dissemination, with new perspectives being integrated across diverse research, scholastic, educational, and training platforms, thus impacting a broad range of stakeholders (34).

An issue considered important in organisational psychology is how perceived corporate social-responsibility goals and values affects employee loyalty, job satisfaction, and motivation, with positive perceptions of corporate social-responsibility activities being associated with increased employee productivity, commitment, and satisfaction (35–38). Some work has taken this further by examining the effects of alignments and misalignments in organisational values and personal values, with alignments having beneficial impacts and facilitating acceptance of positive dynamic change (39–42). The current study is grounded in this tradition, but presents an extension by examining how levels of alignments and misalignments between perceptions of the importance of institutional and personal social-responsibility values affect mental health.

Perceived misalignments, incongruence, and unresolved conflict in beliefs, attitudes, and expectations have been shown to play an important role in many mental health problems (43–47). The extent to which the health and wellbeing of university students is affected by alignments and misalignments between the perceived importance of social-responsibility values held by universities and the personal social-responsibility values held by students has received minimal, if any, research attention. The over-arching aim of the current study was to begin redressing this gap. This is important to understand because it has the potential to not only affect the mental health of students, including countering feelings and perceptions of anti-mattering, but speaks more broadly to how universities nurture social justice principles that endure and extend beyond the institution. We examined personal and perceived social-responsibility values overall, in addition to nine domains of social-responsibility which were derived, *a priori*, from the concept of social justice (30), United Nations Social and Environmental Standards (48), and informed by current concerns about global unrest and potential destabilisation.

It mut be emphasised from the outset that the current study was framed as a ‘proof of concept’ study and somewhat exploratory in nature. Nevertheless, there were two specific research questions. First, which domains of social-responsibility did participants rate as being most important a) to them personally, and b) to their university, and what were the differences between these two perspectives? Second, to what extent were disparities between the importance given to social-responsibility domains from a personal perspective and the importance ascribed to the university, associated with common mental health problems (i.e., depression, anxiety, and stress), both directly and indirectly, with indirect pathways being mediated by perceptions of anti-mattering?

## 2 Methods

### 2.1 Design

The design was cross-sectional (49). Data were collected at one time-point, in two waves spanning the 2023–2024 and 2024–2025 academic years. The difference between ratings of personal importance to the participants and perceived importance to the university, for social-responsibility overall, and for nine domains of social-responsibility were the predictor variables. The mediator variable was anti-mattering perceptions. The outcome variable was a composite measure of depression, anxiety, and stress (depression-anxiety-stress).

### 2.2 Participants

Non-probabilistic convenience sampling (50) was used to recruit students registered at a large UK, Russell Group university. The inclusion criterion was any student registered at the host institution. There were no exclusion criteria. Participants were not reimbursed for their time, other than first- and second-year BSc Psychology students who received four mandatory course credits for participating, equating to 60 minutes work.

There are no formal power calculations available for mediation analysis (Hayes, 2022) but requisite sample sizes were estimated using Monte Carlo simulation modelling (51, 52). From the simulation models, for.8 power, the minimum estimated sample sizes needed for a medium effect size for paths a and b were 78 and 71 for percentile bootstrapping and bias corrected bootstrapping procedures respectively.

### 2.3 Measures

#### 2.3.1 Demographics

Demographics questions included age in years; ethnicity; self-identified gender identity; type of student (undergraduate [UG], postgraduate taught [PGT], MPhil, PhD, professional doctorate); discipline or programme/course studied; year of study; full or part-time status; UK or non-UK status; native English speaker; and self-identified minority status (*“Please tell us to what extent you perceive yourself to be someone of minority status”*).

#### 2.3.2 Depression anxiety stress scale

Depression anxiety stress scale: DASS-21 (53) uses 21 items to measure depressed mood (e.g*., “I felt down-hearted and blue”*), anxiety (e.g., *“I found myself getting agitated”*), and stress (e.g., *“I found it hard to wind down”*) in the past month with a 4-point scale ranging from 0 to 3 (0 - Did not apply to me at all; 1 - Applied to me to some degree, or some of the time; 2 - Applied to me to a considerable degree, or a good part of time; 3 - Applied to me very much, or most of the time). High reliability coefficients have been reported for the total scale (r=.93) (54). The alpha reliability of the DASS total scores for this study was.93.

#### 2.3.3 The anti-mattering scale

The anti-mattering scale (55) is a brief five item scale developed to capture feelings and perceptions of not mattering and marginalisation. The scale captures an overall sense of mattering (Item 1: “*How much do you feel like you don’t matter?”*), self-appraisals of being insignificant (Item 2: *“How often have you been treated in a way that makes you feel like you are insignificant?”*) and self-appraisals of being invisible (item 3: *“To what extent have you been made to feel like you are invisible?”*), together with appraisals of other people’s perceptions (*Item 4: How much do you feel like you will never matter to certain people?; Item 5: How often have you been made to feel by someone that they don’t care about what you think or what you say?”*). Using a 4-point scale from 0 (“Not at all”) to 3 (“A lot”) participants are asked to respond to each question. The reliability coefficients in student samples tend to exceed.75 (53), and was.93 for the current sample.

#### 2.3.4 The social-responsibility importance rating

The social-responsibility importance rating scale comprises 31 items using a one-dimensional scale of 1 (low importance) to 10 (high importance) to rate the perceived importance of different domains of social-responsibility (e.g., *Cultural, ethnic and religious communities; Economic & financial parity*) to the participant personally, and to their university. The scale was generated and tailored, a-priori, specifically for this study based on five sources, three of which involved consultations with students:

i. the concept of social justice framed in the context of mental health (30)
ii. United Nations Social and Environmental Standards encompassing human rights, gender equality, inclusivity, cultural heritage, and climate change and environmental sustainability (48)
iii. experiences of extensive and sustained undergraduate and postgraduate (taught and research) advising, teaching, and supervising
iv. informal consultations with masters level students
v. current issues of relevance to students reflecting global and societal unrest and potential destabilisation.

The United Nations framework provided key elements of social and environmental standards which came into effect in January 2021, and were organised into (A) Programming Principles and (B) Project-level standards. Further information is available from the UN website (48), but in brief, demarcate Principles of i. Leave No One Behind, ii. Human Rights, iii. Gender Equality and Women’s Empowerment, iv. Sustainability and Resilience, and v. Accountability, and Project-Level Standards of Biodiversity Conservation and Sustainable Natural Resource Management; Climate Change and Disaster Risks; Community Health, Safety and Security; Cultural Heritage; Displacement and Resettlement; Indigenous Peoples; Labour and Working Conditions; Pollution Prevention and Resource Efficiency.

Based on these five different sources, including the three sources of student input, the items were generated to fall-into nine domains of social-responsibility which were *Marginalised groups (3 items); Gender identity and sexual orientation (3 items); Health, wellbeing & personal development (6 items); Human and animal rights, and ethical principles (6 items); Cultural, ethnic and religious communities (4 items); Economic & financial parity (3 items); Environmental sustainability (2 items); Social capability and social parity (2 items)*; and *Wars & Global conflict (2 items).* It should be noted that in the 2024–2025 wave of data collection item 13 was changed from *Financial equality and wealth distribution* to *Financial equality and wealth distribution for students*, and item 31 (*A fair distribution of wealth across society*) was added to reflect global economic inequalities. This was because an initial analysis of open responses probing social-responsibility values indicated that the majority of participants felt financial pressure from being students meaning that including one item that explicitly reflected the specific university/student context seemed appropriate. Cronbach’s alpha for all 31 items=.96. The items used together with their classification into nine social-responsibility domains, together with participant instructions can be found in **Supplementary Table S1** in **Supplementary Materials**.

##### 2.3.4.1 Content validity of social-responsibility domains: an analysis of open response questions

Participants were also invited to respond to several Social-Responsibility open response questions, two of which we examined in more detail to determine the extent to which the nine domains of social-responsibility resonated with the student participants, and to lend validity to the Social-Responsibility Importance rating scale. Specifically, we addressed questions of i. were the domains of social-responsibility represented in the scale similar to the domains spontaneously generated by students, and ii. did students endorse domains of social-responsibility not represented in the scale? The two items were:

1. Please tell us more about any types of social-responsibility that are particularly important to you, and why?
2. Please tell us more about any types of social-responsibility that seem particularly important to your university, and why?

In examining these two social-responsibility open questions qualitatively, we focused on any new social-responsibility domains that participants noted. For question 1 (personal importance) the vast majority of responses (N=89) echoed the nine identified domains with some reflecting the importance of social-responsibility across many explicit domains (e.g., *“human rights, ethics, equality and anti-racism because they are issues of such extreme importance that they should be witnessed on a regular basis, whether that’s in the news or in person” ID=2*), or the importance to them of social-responsibility broadly (e.g., *“I think people have an obligation to do any small bit in their life where they can for all of the above. Even a small action can ripple out” ID=164*). There were four new social domains identified by four separate participants of i. Providing a better education for youngsters *(“Educate youngsters better” ID=3*), ii. Social-responsibility in corporate law *(“social responsibility in corporate law” ID=18)*, iii. Ensuring fair and safe working environments *(“Fair Labour Practices. Ensuring a fair and safe workplace is critical to protecting employee rights and benefits. Fair labour practices contribute to an upright and productive work environment that promotes the overall well-being of society” ID=36)* and iv. Social-responsibility in everyday actions *(“I feel that the issues of social responsibility that are most important to me are those that affect or come up in my everyday life the most ID=5)*.

For question 2 (university social-responsibility), responses (N=88) reflected the perceived breadth of commitment to social-responsibility by the university in general *(*e.g., *“All of them. The university needs to be a place everyone feels safe welcome and included” ID 123; “All types of social-responsibility are important to the university” ID=145*) or by explicitly mentioning specific domains with cultural diversity and integration, climate action, mental health, gender equality, financial parity, and social justice being identified frequently. There were four new social-responsibility domains identified which were i. Ensuring employability *(e.g., “getting a job” ID=136*), ii. Personal identity *(e.g., “Universities seem to take personal identity very seriously” ID=36)*, iii. Altruism in the context of mental *health (e.g., “Looking after friends and their mental health” ID=93)*, and iv. Encouraging a work-life balance (e.g., *“support mental health and work life balance whilst also helping us strive to do our very best at uni” ID=192*). Overall, these open question response data demonstrated that the Social-Responsibility Importance rating scale reflected domains which were meaningful to students personally, and also with respect to their perceptions of their university. Examples of direct quotes to each of these two open response questions can be found in the **Supplementary Table S2** in the **Supplementary Materials**.

### 2.4 Procedure

Participants were invited to take part in the study primarily via SONA systems (https://www.sona-systems.com/) which is an online platform used to advertise research projects to psychology undergraduates, together with wider online announcements to student cohorts. Interested participants followed a link to an online survey via Qualtrics. After reading the participant information sheet and providing informed consent digitally participants completed the online survey. Responses were completely anonymous. The same order of measures was used across participants. Signposting to mental health resources was presented at the beginning and end of the survey. Ethical approval was granted by the University Research Ethics Committee 3, Reference 2024-18434-32480.

### 2.5 Statistical analyses plan

Associations between variables were analysed with Pearson’s Product Moment correlation coefficients with bootstrapping at 5000 iterations, because some variables were not normally distributed (56). Differences in importance ratings between the nine social-responsibility domains for the self, versus those ascribed to the university were analysed with a MANOVA. A series of linear regression models were used to test indirect (mediated) and direct paths between the predictors (i.e., the difference between personal and institutional importance ratings of social-responsibility overall, and for nine domains of social-responsibility), and the outcome variable (i.e., DASS-21, Depression-Anxiety-Stress Scale scores) with the mediator of anti-mattering (total score plus scores for each of 5 items), using template 4 of the Process algorithm (57). The alpha level was.05, with Bootstrapped 95% percentile Confidence Intervals (CIs) at 5000 iterations. It must be noted that the design was cross-sectional which means that neither temporal precedence nor causality can be inferred from significant indirect effects in mediation models (58).

## 3 Results

### 3.1 Participant characteristics

The mean age of participants was 19.21 (SD=2.03; N=171), and all studied BSc (Hons) Psychology. The majority of participants identified as female/woman; were first year undergraduate students; studied full-time; were registered as UK students; and were native English speakers. About 50% of participants identified as White/White British and 45% identified as being of minority status at least to some extent. (Please see **Supplementary Table S3** in **Supplementary Materials**).

### 3.2 Research question 1: which domains of social-responsibility did student participants rate as being most important a) to them personally, and b) to their university, and what were the differences between these two perspectives?

**Table 1** presents the mean importance ratings across the nine domains of social-responsibility, from the perspectives of importance to the self, and importance to the university. The domain that was rated highest in terms of perceived personal importance was *Health, wellbeing and personal development*, and the lowest was *Wars & Global conflict.* In terms of perceived importance to the university, the highest rated domain was also *Health, wellbeing and personal development*, with the lowest being *Economic and financial parity overall*. All domains were rated as being significantly more important to the self, compared to ratings of the perceived importance ascribed to the university [F (9,163)=22.26, p <.0001, partial eta squared=.55; with partial eta squared ranging from.38 for *Economic and financial parity overall* to.015 [not significant] for *Environmental sustainability*].

**Table 1:** Descriptive statistics (Mean, Standard deviation [SD]) for i. personal and university social-responsibility (SR) importance ratings; ii. differences in those importance ratings between the self and the university, iii. mental health problems (DASS), and iv. anti-mattering scores.

N=171	Personal		University		Difference in SR		Correlation coefficients	
	Mean	SD	Mean	SD	Mean	SD	DASS	Anti-mattering
Marginalised Groups	8.27	1.61	7.31	1.99	0.97	2.08	.23**	.21**
Gender identity & sexual orientation	8.12	2.06	7.55	2.12	0.57	2.59	.20**	.19*
Health, wellbeing & personal development	8.54	1.21	7.60	1.59	0.94	1.68	.30**	.26**
Human and animal rights & ethical principles	8.14	1.30	6.74	1.98	1.40	2.18	.24**	.20**
Cultural, ethnic & religious communities	7.91	1.52	7.56	1.70	0.35	1.86	.26**	.20**
Economic and financial parity overall	8.09	1.62	6.09	2.41	2.00	2.55	.24**	.22**
Environmental sustainability	7.83	1.87	7.54	1.94	0.29	2.52	.10	.15
Social parity & development	8.03	1.74	6.74	2.18	1.29	2.34	.23**	.19*
Wars & Global conflict	7.54	1.65	6.94	2.14	0.60	2.40	.14	.15

Pearson’s Product Moment correlation coefficients have been provided between the difference scores and the DASS and anti-mattering scores with bootstrapping applied at 5000 iterations.

* p<= .05; ** p<= .01.

**Figure 1:**
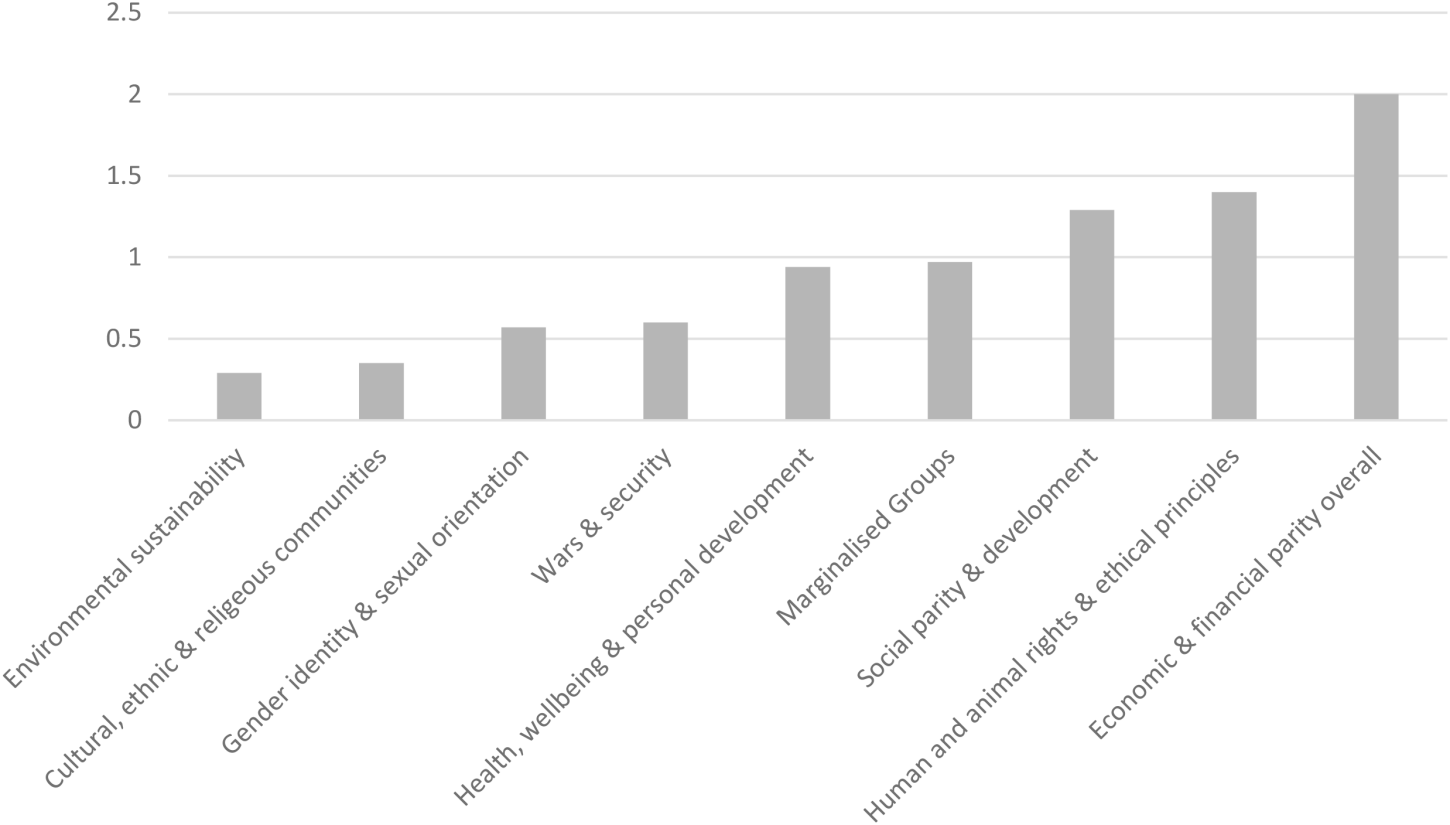
Differences in social-responsibility importance ratings across 9 domains held personally (self) minus those perceived to be held by the university.

As can be seen from **Figure 1** and **Table 1**, the greatest difference, or disparity, between the importance of personal social-responsibility values versus those perceived to be held by the university was for the domain of *Economic and financial parity overall*. The domain of *Environmental sustainability* evidenced the least disparity.

### 3.3 Research question 2: to what extent were disparities between the importance given to social-responsibility domains from a personal perspective and the importance ascribed to the university, associated with common mental health problems (depression-anxiety-stress), both directly and indirectly, with indirect pathways being mediated by perceptions of anti-mattering?

Each item of the DASS was scored 0 – 3, giving a possible range of 0 to 63, and a mean of 22.24 (SD=13.08). The mean for an undergraduate student sample was recently recorded as 25.22 and for non-clinical adult samples as 17.80 and 18.86 (59). The possible range of scores for the anti-mattering scale was 5 – 20, with a mean of 9.81 (SD=3.24). In a sample of Canadian undergraduate students a mean of 11.17 was recently documented (60). Differences between social-responsibility importance scores for self, versus those attributed to the university were significantly correlated with mental health problems (DASS) and anti-mattering across all domains, apart from *Environmental sustainability* and *Wars & Global conflict* (see **Table 1**).

As shown in **Table 2**, significant indirect effects, mediated by anti-mattering perceptions, were evident for the relationships between difference in social-responsibility importance ratings (overall, and for 8 of the 9 different domains of social-responsibility) and depression-anxiety-stress scale scores. Of note, and in accord with correlational analyses, the mediated pathway when the difference in social-responsibility importance ratings for *Wars & Global conflict* was the predictor variable was not significant. There were significant direct effects for the overall difference in social-responsibility importance ratings, and for domains of *Health, wellbeing & personal development; Human and animal rights & ethical principles; Cultural, ethnic & religious communities; Economic & financial parity overall*; and *Social parity & development.* Direct effects were not significant for *Marginalised groups*, *Gender identity and sexual orientation, Environmental sustainability, and Wars and global conflict* social-responsibility domains.

**Table 2:** Direct and indirect (mediated) effects for a pathway linking predictor variables of differences between the personal importance given to social-responsibility (SR) domains and those perceived as being held by the university, and the outcome variable of mental health problems (measured by DASS), mediated by anti-mattering perceptions.

N=171	Direct effect c’					Indirect effect				Completely standardised indirect effect			
Predictor variable			CIs					CIs				CIs	
Differences in social-responsibility importance	Effect	SE	5%	95%	c’-CS	Effect	SE	5%	95%	Effect	SE	5%	95%
Overall Difference score	1.04*	.48	.10	1.98	.15	.73*	.26	.24	1.26	.11*	.04	.03	.18
*Marginalised groups*	0.81	.43	-.03	1.66	.13	.61*	.22	.19	1.06	.10*	.04	.03	.17
*Gender identity & sexual orientation*	0.57	.34	-.11	1.25	.11	.44*	.19	1.00	.83	.09*	.04	.02	.16
*Health, wellbeing & personal development*	1.41*	.53	.36	2.46	.18	.89*	.29	.37	1.48	.11*	.04	.05	.19
*Human and animal rights & ethical principles*	0.88*	.41	.08	1.68	.15	.54*	.22	.11	1.00	.09*	.04	.02	.16
*Cultural, ethnic & religious communities*	1.18*	.47	.24	2.11	.17	.63*	.26	.14	1.16	.09*	.04	.02	.16
*Economic & financial parity overall*	0.75*	.35	.06	1.43	.15	.50*	.18	.16	.89	.10*	.04	.03	.17
*Environmental sustainability*	0.15	.35	-.54	.85	.03	.37*	.20	.01	.78	.07*	.04	.004	.15
*Social parity & development*	0.81*	.38	.06	1.56	.15	.48*	.20	.10	.89	.09*	.04	.02	.16
*Wars & Global conflict*	0.38	.37	-.35	1.11	.07	.38	.22	-.04	.84	.07	.04	-.008	.15

Pathways involving differences in SR importance ratings overall, and for each of 9 social-responsibility domains, have been shown. Completely standardised indirect effects have also been shown along with standard direct effects (c’-cs). Bootstrapping was applied at 5000 iterations. (SE, Standard Error; CI, Confidence Interval.) Significant pathways have been indicated with an asterisk.

* p<= .05.

### 3.4 Planned exploratory analyses

**Figure 2:**
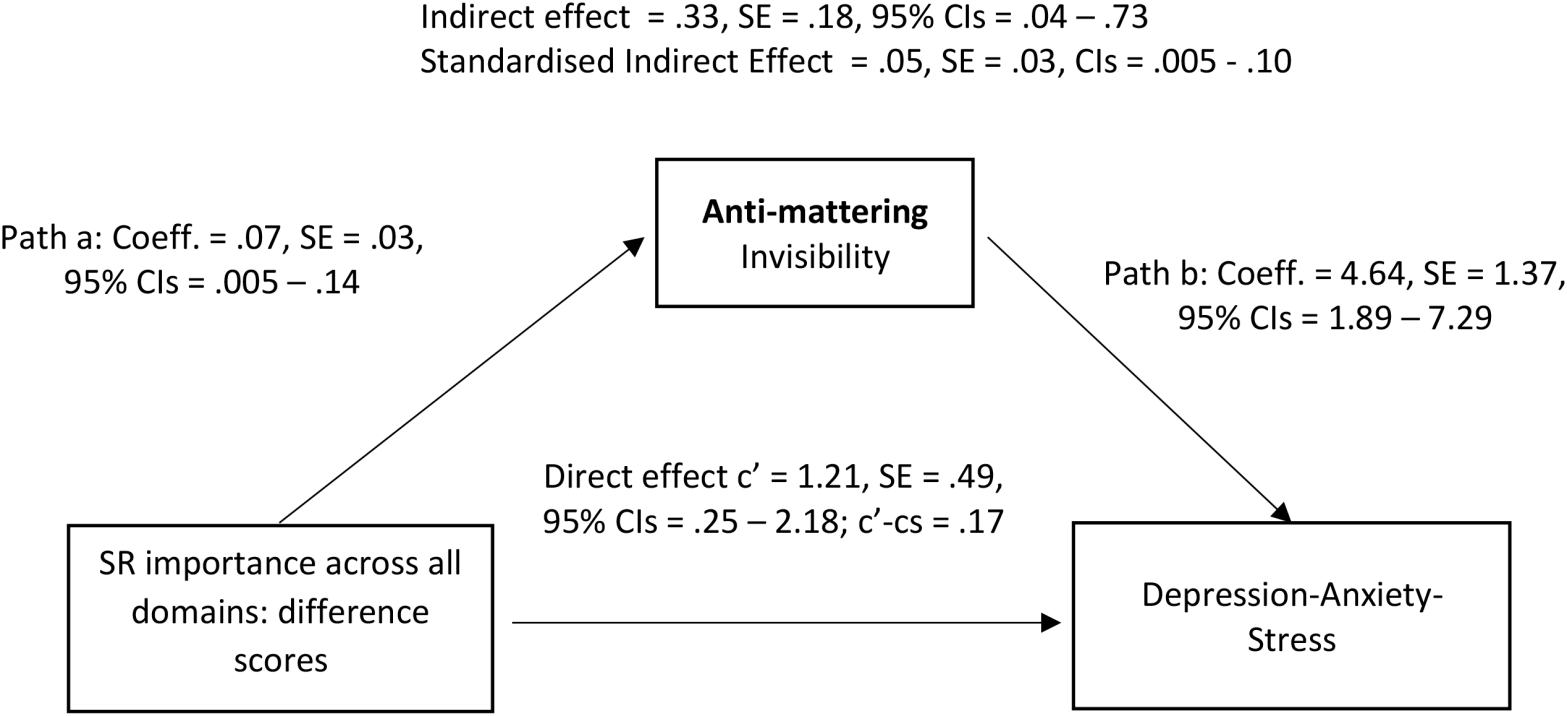
Significant direct and indirect pathways between the disparity in the importance of social-responsibility perceived from personal (self) versus perceived university perspectives (predictor) and DASS depression-anxiety-stress total scores (outcome), with the invisibility component of anti-mattering acting as a mediator. Non-standardised and completely standardised indirect effects have been provided along with effect size (c’=cs) for the direct effect, path c’.

We attempted to gain a more in-depth understanding of the significant mediation effects by repeating the mediation models but with the five different items comprising the anti-mattering scale as parallel mediators. The correlation coefficients between the invisibility anti-mattering item and the not mattering and insignificance anti-mattering items were.57 and.49 respectively (p <.001). The associations between the difference in social-responsibility importance scores and both anti-mattering items was.17 (p <.05). The correlation coefficients between the depression-anxiety-stress scores and invisibility and insignificance were.46 and.38 respectively (p <.001). In the mediation model, the direct effect was significant – the difference in social-responsibility importance scores were associated with depression-anxiety-stress. The only significant mediated, indirect, effect was when anti-mattering item 3 depicting invisibility was the mediator (see **Figure 2**).

Of the nine social-responsibility domains, a significant indirect effect with the invisibility anti-mattering item as the mediator was observed for *Health, wellbeing & personal development* (Direct Effect=1.65, SE=.54, 95% CIs=.58 to 2.72, c’-cs=.21; Indirect Effect=.38, SE=.21, 95% CIs=.04 to.86; Standardised Indirect Effect (SIE)=.05, SE=.03, CIs=.006 to.11), *Cultural, ethnic & religious communities* (Direct Effect=1.25, SE=.48, 95% CIs=.30 to 2.19, c’-cs=.10; Indirect Effect=.31, SE=.17, 95% CIs=.02 to.67; SIE=.04, SE=.02, CIs=.003 to.10), *Economic & financial parity overall* (Direct Effect=.87, SE=.36, 95% CIs=.16 to 1.58, c’-cs=.17; Indirect Effect=.28, SE=.15, 95% CIs=.04 to.61; SIE=.05, SE=.03, CIs=.008 to.12), and *Environmental sustainability* (Direct Effect=.22, SE=.36, 95% CIs=-.50 to.93, c’-cs=.04 [not significant]; Indirect Effect=.23, SE=.13, 95% CIs=.008 to.53; SIE=.05, SE=.03, CIs=.002 to.10). A significant indirect effect was not found for five of the social domains of *Marginalised groups, Gender identity & sexual orientation, Human and animal rights & ethical principles, Social parity & development* and *Wars & Global conflict.*

## 4 Discussion

The overarching aim of the current study was to examine the extent to which misalignments in the perceived importance of social-responsibility to participants personally versus their university would be associated with mental health problems both directly, but also indirectly when mediated by appraisals of not mattering or anti-mattering (6). There were three key findings.

First, importance across all nine domains of social-responsibility was higher when rated from the perspective of the self, as opposed to the perceived importance attributed to the university. The greatest difference was observed for the domain of *Economic and financial parity* and the smallest difference was for *Environmental sustainability*. A large amount of literature has documented the considerable detrimental effect of financial pressures on students’ mental health (61–63). Currently, in the UK, students face serious financial pressures not only because of escalating austerity but also from rising university fees (64). This fits with a growing body of evidence documenting negative effects of austerity on mental health and mental health provision not only within the UK but more broadly, with an important point being that the expectations of young people and their perspectives of their future are somewhat pessimistic as a consequence of austerity measures and policies (65–72). Qualitative responses lent reassurance that the social-responsibility domains were of relevance to students. That said, at the time of writing, instability is acute across geopolitical, economic, and human rights platforms globally meaning that perceptions of needed social-responsibility priorities are likely to change.

The second finding was that disparities in importance ratings between students and those attributed to their university across seven domains of social-responsibility were significantly and positively associated with a composite measure of depression-anxiety-stress. The greater the disparity the more severe were the mental health problems. Studies investigating misalignments between corporate social-responsibility values and those of employees have noted ways in which positive perceptions of alignment can affect staff performance, productivity, cohesion and acceptance of change (39–42). We have expanded this literature by demonstrating the effect of such misalignments on the mental health of students. The two social-responsibility domains which did not evidence these associations were *Wars and global conflict*, and *Environmental sustainability.* The personal importance ratings of these two domains were the lowest which was somewhat surprising in the context of the number of serious conflict situations which were affecting global stability at the time of writing, and also due to significant concerns about climate change which can negatively impact mental health (73, 74). That said, many of the open-ended responses of the students noted the proactive attitude of the university with regard to climate change which may have countered climate change anxieties. During the reviewing process, one reviewer pointed out that misalignments in social-responsibility importance may not be inherently negative, with misalignments in certain domains being either less impactful for students or not imply any cause for concern. Hence, it seems important to explicitly examine participant’s own views of such misalignments in future work which we were not able to do in the current study.

The third finding expanded on the second, in that although there was a direct relationship between differences in social-responsibility importance ratings (personal minus university) and mental health problems, there was also an indirect, mediated, effect with feelings and perceptions of anti-mattering being the mediator. This indirect effect meant that social-responsibility misalignment scores were associated with anti-mattering scores, and anti-mattering scores were associated with depression-anxiety-stress. The detrimental effects of anti-mattering, or not mattering, on mental health in the student body have been documented, at least to an extent (4, 9, 24, 25, 27). When examining the different components of anti-mattering, it was perceptions of invisibility (*“To what extent have you been made to feel like you are invisible?”*) rather than insignificance which were key. In-depth qualitative research is now needed to explore from where this sense of invisibility emanates. Reflections from the authorship team, one of whom was a university student, highlighted a need to understand in university contexts how a sense of invisibility may i. fluctuate; ii. accumulate from numerous sources; iii. be affected by perceptions of not fitting-in, for example, with respect to culture, religion, ethnicity, gender identity, and sexual identity; iv. relate to the size of both the institution and the city (‘small fish in a large pond’); v. be driven by a sense of inferiority with respect to finance and financial back-ground; and vi. be related to perceptions that universities do not integrate enough personalisation into developing their goals. It was also felt important to better determine the extent to which perceptions of invisibility reflected an active attempt by students to make their voices heard, but felt that these active attempts were then thwarted, dismissed, or ignored by the institution.

### 4.1 Limitations

A number of limitations of the current study warrant discussion. First, the Social-Responsibility Importance ratings scale was constructed specifically for this study and was deliberately one-dimensional in only asking participants about perceptions of importance. The descriptors for each item were also intentionally as simple as possible. This seemed appropriate given that the work was framed as a ‘proof of concept’ study. Nevertheless, categorising each of the items into nine social-responsibility domains was somewhat artificial in that some items could suitably fit under more than one domain. Countering this concern, at least to a degree, the overall difference score across all the social-responsibility domains evidenced the same pattern as eight out of nine of the specific social-responsibility domains, and the one domain (*Wars & Global conflict*) that did not evidenced a significant mediation effect comprised only two items. Finally, responses to two open response questions probing social-responsibility lent reassurance that the scale was of relevance to participants, and was comprehensive. There is clear, and necessary, scope in further work in this area for developing the depth of individual’s social-responsibility perceptions beyond the one-dimensional construct of importance using convergent qualitative and quantitative techniques.

Second, only one UK institution was involved in this study with all participants studying psychology and the majority of participants being home students, limiting generalisability. Social-responsibility values may differ considerably between UK and non-UK universities. Furthermore, the institution is part of the Russell Group (https://www.russellgroup.ac.uk/our-universities) meaning that students might be expected to be ethnically and culturally homogenous, to largely come from a privileged background, and to insufficiently represent marginalised groups. Although the university is Russell Group, it is situated in Northern England, known for its working-class and industrial heritage. Furthermore, in the current study, 50% of the participants identified as not being white and approximately 45% indicated that they felt they were of minority status attesting to the heterogeneity of the sample, at least to some extent.

Third, and relatedly, the participants received mandatory course credits in return for participation. Students in the host institution are offered many studies in which to participate from a wide range of areas in psychology (e.g., qualitative, mental health, neurophysiology, language, cognitive experimental). This means that they were unlikely to have felt coerced into participating in this particular study. That said, they were quite likely to have been motivated to take part because they were interested in this area. Hence, they may have been more motivated compared to other similarly aged young adults.

Fourth, the design was cross-sectional meaning that neither temporal precedence nor causality can be inferred. A related design issue is that the impact of participant’s history of, or current, mental health problems at clinical severity levels could not be considered or statistically controlled for.

Finally, perceptions of the importance of social-responsibility values especially when considered in tandem with mattering and anti-mattering appraisals, require a convergent methods approach as the work develops which was beyond the scope of the current study. The findings that we present provide a starting point, and a platform for in-depth qualitative follow-up work which can better probe the complexities surrounding many social-responsibility domains, such as war and global conflict, and the impact of generative artificial intelligence.

In conclusion, it is important to understand ways of improving mental health from both individual and systemic perspectives. One way of doing this is to develop authentic alignments between the social-responsibility agendas of institutions and a broad range of stakeholders that go beyond imposing social-responsibility values that can seem akin to vacuous ‘tick-box’ exercises. It is essential to genuinely listen to, to actively understand, and to implement measures to embrace the social-responsibility values expressed by students, in particular. Furthermore, where alignments seem challenged or distant, it is vital to counter the perceptions of stakeholders that they do not matter and that they are invisible.

## Data Availability

The raw data supporting the conclusions of this article will be made available by the authors, without undue reservation.
