# Supplementary material for: Anti-mattering mediates the relationship between social-responsibility misalignments and mental health problems in young people
Source: Front Psychiatry. 2025 Oct 15;16:1639802. doi: 10.3389/fpsyt.2025.1639802 (PMC12569645; doi:10.3389/fpsyt.2025.1639802)
Supplement: Supplementary file 1 [file DataSheet1.pdf]

Supplemental Table S1

Instructions together with the 31 items comprising the Social Responsibility Importance rating scale and the 9 social responsibility domains inro which the items were categorised.

Instructions:

There are many different types of social responsibility, sometimes about individuals and sometimes about communities, organisations, and/or systems. Here is a list of some of the different types of social responsibility. Using a scale of 1 (not important) to 10 (very important) please tell us i. how much each is important to you, and ii. how much each seems important to your university.

	Item	*Social Responsibility Domain*
1	Anti-racism	Marginalised groups
2	Personal capability and development	Health, wellbeing, and personal development
3	Community integration and engagement	Cultural, ethnic and religious communities
4	Anti-bullying	Health, wellbeing, and personal development
5	Human rights and human ethics	Human and animal Rights, and ethical principles
6	Global unrest and aggression	Wars and global conflict
7	Mental health	Health, wellbeing, and personal development
8	Anti-ableism	Marginalised groups
9	Political activism	Human and animal Rights, and ethical principles
10	Environmental sustainability	Environmental sustainability
11	Cyber-security and AI	Wars and global conflict
12	Intellectual inclusivity	Human and animal Rights, and ethical principles
13	Financial equality and wealth distribution for students	Economic and financial parity:
14	Community investment	Cultural, ethnic and religious communities
15	Trans-rights	Gender identity & sexual orientation
16	Political transparency	Human and animal Rights, and ethical principles
17	Anti-ageism	Marginalised groups
18	Physical health	Health, wellbeing, and personal development
19	Social and economic parity across nations	Social capability and social parity
20	Animal rights	Human and animal Rights, and ethical principles
21	Gender equality and identity	Gender identity & sexual orientation
22	Ethical decision making	Human and animal Rights, and ethical principles
23	Physical security	Health, wellbeing, and personal development
24	Sexual orientation	Gender identity & sexual orientation
25	Climate action	Environmental sustainability
26	Religious equality and identity	Cultural, ethnic and religious communities
27	Economic transparency and fair trade	Economic and financial parity:
28	Neurodiversity awareness	Health, wellbeing, and personal development
29	Cultural, equality and identity	Cultural, ethnic and religious communities
30	Social capability and development	Social capability and social parity
31	A fair distribution of wealth across society	Economic and financial parity

Marginalised groups 3 items: 1, 8, 17
Gender identity & sexual orientation 3 items: 15, 21, 24
Health, wellbeing, and personal development 6 items: 2, 4, 7, 18, 23, 28
Human and animal Rights, and ethical principles 6 items: 5, 9, 12, 16, 20, 22
Cultural, ethnic and religious communities 4 items: 3, 14, 26, 29
Economic & financial parity 3 items: 13, 27, 31
Environmental sustainability 2 items: 10, 25
Social capability and social parity 2 items: 19, 30
Wars and global conflict 2 items: 6, 11

Supplemental Table S2

Examples of direct quotes to the two questions used to assess to what extent the nine dimensions of social-responsibility reflected student participant's views as expressed in their own words. This illustrative corpus was chosen at random as long as participants had provided responses to both questions.

	PLEASE TELL US MORE ABOUT ANY TYPES OF SOCIAL-RESPONSIBILITY THAT ARE PARTICULARLY IMPORTANT TO YOU, AND WHY?	***PLEASE TELL US MORE ABOUT ANY TYPES OF SOCIAL-RESPONSIBILITY THAT SEEM PARTICULARLY IMPORTANT TO YOUR UNIVERSITY, AND WHY?***
**1**	Politics. Reinvestment into the community. LGBTQ+ Rights	Seem to care about climate and LGBTQ+ rights
**2**	human rights, ethics, equality and anti-racism because they are issues of such extreme that it witnessed on a regular basis, whether that's in the news or in person	gender equality - I have seen advertisements for equality everywhere across the university and SU [student union]
**3**	I believe all possible types of social responsibility are important, especially climate action because of the recent rise of the cases where people were in a huge danger like earthquake in Morocco, flooding in the U.K., U.S. and other countries. Also, human rights and mental health seemed to be on the agenda,	Climate and development, I assume because it is one of the aims of the university to develop students and help to find their desirable career
**4**	mental health I really want to help with this, any discrimination against a minority group e.g. racism, trans, sexual orientation, women make me feel so angry because it feels so unjust to me. environment is also important as we have been given a beautiful world and we shouldn't throw it away out of greed	they seem very good with the multicultural nature of the university and seem to have a lot of societies set up for international students
**5**	I particularly am interested in any areas regarding minority groups and inclusivity and equality. I believe that everyone has a right to be themself regardless or race, sex, religion, etc., and that they shouldn't be stigmatised, discriminated against or hated on because of who they are.	There seems to be a lot of activism around environmental issues, race and religious issues as well as political issues
**6**	Economic transparency	Mental health
**7**	Everything related to politics is very important to me because I feel that is where the difference begins. Also, those related to equality between gender, religions, sexual orientation, etc., because differences should not be made on this basis, but on the person's abilities in the respective field. Last but not least, animal rights because I have been surrounded by animals for as long as I know myself.	Those related to equality between gender, religions, sexual orientation, etc. because at this University there are many people who are part of various minorities.
**8**	Animal rights and spreading awareness about poverty	Anti racism as a lot of diversity at [DELETED].
**9**	None really. I see an issue today with a lack of care for physical health and mental health in quite a lot of people, but I feel like poor physical health can heavily contribute in some to poor mental health	focus on being proud of your sexuality/orientation and what you identify as more recently.
**10**	Equality and inclusivity of all types, because I think it is extremely important that people from all groups have equal access to opportunities	The main thing that seems to be talked about it climate action
**11**	Human rights and human ethics and animal rights are particularly important to me	Personal capability and development and mental health seem to be particularly important to the university
**12**	climate change is important as it effects the whole world	I think ensuring minority groups are not excluded is important
**13**	environmental and sustainability issues, because without addressing climate change the other things will no longer be important in a number of years	Certain issues seemed to be a lot more talked about at the university, for example just from thinking about what I see on the [DELETED} Instagram there is a lot about mental health, cultural identities and cost of living/ finance issues

**14**	Human ethics- some countries are still really not like 1st world countries and I think that should really start to evolve.	Cultural inclusivity.<br>Looking out for struggling individuals- mentally supporting them.
**15**	Anti-racism, and human rights are extremely important to me, as well as many forms of equality. These are important as I believe they play a serious role in today's world and causes significant conflicts and damage.	Anti-racism, anti-bullying, and sexual orientation seem particularly important to the university as they are factors that directly effect the university and the students that attend.
**16**	Gender equality as I have seen how women are still viewed as inferior in society.	They talk a lot about mental health and provide a lot of information and services to help with that.
**17**	Racism, financial inequality and wealth distribution, physical and mental health of people in society.	Cultural integration, gender equality, cyber-security - the things I notice they put effort into. Can't recall exactly where but I see it around.
**18**	I think racist and homophobic issues are of upmost importance as people get physically and verbally abused because of these issues. I also think that sexism and sexual abuse/harassment are important issues as it is extremely prevalent in today's society	mental health, because they often send e-mails/ send relief to chat
**19**	Any kind of equality achievement is important to me because everyone should be treated as equals	I feel like the university takes social responsibility for a lot of these things as they concern us as students and our futures
**20**	Human rights and human ethics and animal rights are particularly important to me	Personal capability and development and mental health seem to be particularly important to the university

Supplemental Table S3

Demographic characteristics of the sample of participants (N = 171).

	NUMBER	PERCENTAGE
**GENDER**		
FEMALE	138	80.7%
MALE	20	11.7%
WOMAN/CIS-GENDER WOMAN	8	4.7%
MAN/CIS-GENDER MAN	1	0.6%
AGENDER	1	0.6%
NON-BINARY	1	0.6%
**ETHNICITY**		
WHITE/WHITE BRITISH	85	49.7%
ASIAN	59	34.5%
EUROPEAN	11	6.4%
MIXED	8	4.7%
ARABIC	1	0.6%
LATIN-AMERICAN	1	0.6%
**YEAR OF UNDERGRADUATE STUDY**		
1	131	76.6%
2	39	22.8%
3	1	0.6%
**FULL OR PART-TIME**		
FULL	169	98.8%
PART-TIME	2	1.2%
**UK OR NON-UK**		
UK	124	72.5%
NON-UK	47	27.5%
**NATIVE ENGLISH SPEAKER**		
YES	129	75.4%
NO	42	24.6%
**MINORITY STATUS (SELF-IDENTIFIED)**		
NO	73	42.7%
YES	53	31.0%
TO SOME EXTENT	17	9.9%
TO A MODERATE EXTENT	4	2.3%
TO A LARGE EXTENT	4	2.3%
